# Seed bank dynamics govern persistence of *Brassica* hybrids in crop and natural habitats

**DOI:** 10.1093/aob/mcu213

**Published:** 2014-11-30

**Authors:** Danny A. P. Hooftman, James M. Bullock, Kathryn Morley, Caroline Lamb, David J. Hodgson, Philippa Bell, Jane Thomas, Rosemary S. Hails

**Affiliations:** ^1^Centre for Ecology and Hydrology, Benson Lane, Wallingford OX10 8BB, UK, ^2^School of Biosciences, University of Exeter, Cornwall Campus, Penryn TR10 9EZ, UK and ^3^National Institute of Agricultural Botany, Huntingdon Rd, Cambridge CB3 0EL, UK

**Keywords:** *Brassica napus*, *Brassica rapa*, demography, fitness, gene flow, genetically modified crops, hybridization, introgression, management, crop rotation

## Abstract

**Background and Aims** Gene flow from crops to their wild relatives has the potential to alter population growth rates and demography of hybrid populations, especially when a new crop has been genetically modified (GM). This study introduces a comprehensive approach to assess this potential for altered population fitness, and uses a combination of demographic data in two habitat types and mathematical (matrix) models that include crop rotations and outcrossing between parental species.

**Methods** Full life-cycle demographic rates, including seed bank survival, of non-GM *Brassica rapa* × *B. napus F*_1_ hybrids and their parent species were estimated from experiments in both agricultural and semi-natural habitats. Altered fitness potential was modelled using periodic matrices including crop rotations and outcrossing between parent species.

**Key Results** The demographic vital rates (i.e. for major stage transitions) of the hybrid population were intermediate between or lower than both parental species. The population growth rate (*λ*) of hybrids indicated decreases in both habitat types, and in a semi-natural habitat hybrids became extinct at two sites. Elasticity analyses indicated that seed bank survival was the greatest contributor to λ. In agricultural habitats, hybrid populations were projected to decline, but with persistence times up to 20 years. The seed bank survival rate was the main driver determining persistence. It was found that *λ* of the hybrids was largely determined by parental seed bank survival and subsequent replenishment of the hybrid population through outcrossing of *B. rapa* with *B. napus*.

**Conclusions** Hybrid persistence was found to be highly dependent on the seed bank, suggesting that targeting hybrid seed survival could be an important management option in controlling hybrid persistence. For local risk mitigation, an increased focus on the wild parent is suggested. Management actions, such as control of *B. rapa*, could indirectly reduce hybrid populations by blocking hybrid replenishment.

## INTRODUCTION

Gene flow and hybridization between crops and their wild relatives is common and has been so since the beginning of agriculture (Ellstrand, 2003). A substantial number of molecular studies have revealed permanent incorporation – introgression – of crop genes into the genomes of wild and weedy relatives (Kwit *et al*., 2011; Ellstrand *et al*., 2013). In the case of planting of genetically modified (GM) crops this incorporation of (trans)genes into wild relatives is generally considered as undesirable (Chandler and Dunwell, 2008; Schierenbeck and Ellstrand, 2009) and has been found in several cases (Ellstrand *et al*., 2010). This includes the occurrence of transgenic hybrids among *Brassica* species (Warwick *et al*., 2008).

Gene flow between crops and wild relatives does not per se constitute a risk to the environment, but could alter the dynamics of plant populations, changing their population growth rates (‘fitness’) and persistence both inside and outside an agricultural environment. Such increased fitness might lead to environmentally damaging changes in agricultural management with regard to herbicide use and weeding practices (Hawes *et al*., 2003), or changes in overall structure and function of ecosystems (Chandler and Dunwell, 2008; Kwit *et al*., 2011). For transgenes, hybrid populations could also act as a temporal bridge between successive crops through outcrossing (Squire *et al*., 2011). Crop genes have been found to persist for several years in and around agricultural fields through introgression into wild relatives (Ellstrand *et al*., 2013) or through the persistence of volunteers and plants growing outside the agricultural fields, i.e. feral plants (Pivard *et al*., 2008; Schafer *et al*., 2011; Devos *et al*., 2012).

Current guidelines on the environmental risk assessment of growing GM plants in the European Union (EFSA, 2010) emphasize that there might be environmental consequences both inside and outside agricultural habitats that result from changes in population dynamic caused by gene flow among crops and wild relatives. Similar points have been made in North America (Snow *et al*., 2005). Therefore, assessments need to include a consideration of the dynamics of hybrids in both agricultural and semi-natural habitats.

Quantification of the effects of gene flow of GM crops involves both the potential to produce viable offspring (compatibility) and the consequences of the gene flow for population viability, i.e. fitness (Hails and Morley, 2005). The compatibility of crops with many wild relatives is well characterized (Ellstrand *et al*., 2013), including precise estimates of hybridization and flower synchrony rates for *Brassica* (Norris *et al*., 2004; Devos *et al*., 2009). In particular, hybridization between wild turnip (*B. rapa*) and oilseed rape (OSR) *B. napus* has been shown to occur naturally (Wilkinson *et al*., 2003; Jørgensen *et al*., 2009; Luijten *et al*., 2014). The second stage in assessing risks – the consequences for plant population fitness – requires experimental demographic data for hybrids relative to the parental species; see Hooftman *et al.* (2007), Campbell *et al.* (2009) and Snow *et al.* (2010) for examples, and specifically Snow *et al.* (1999), Allainguillaume *et al.* (2006), Jørgensen *et al.* (2009) and Vacher *et al.* (2011) for examples involving *Brassica*. However, most of these studies have not included the full life cycle or have combined data from different experiments, reducing the validity of any predictions to assess fitness in terms of the population growth rate (Hails and Morley, 2005).

Accurate assessment of changes in fitness of hybrids must incorporate several fundamental ecological considerations (Hails and Morley, 2005). First, fitness is an integrated measure of performance over all life stages requiring demographic data for all major stage transitions, i.e. vital rates. Such data are best combined by employing population models (Bullock, 1999). Several studies into crop–wild hybrids have considered above-ground stages (e.g. Hooftman *et al*., 2007; Vacher *et al*., 2011; Yang *et al*., 2011; Cao *et al*., 2014), but none has included simultaneous measures of seed bank survival.

Second, fitness is habitat-dependent and so studies within single environments have only a limited value for risk assessment (Ridley and Ellstrand, 2009; Snow *et al*., 2010). In particular, crop–wild relative hybrids are usually studied in agricultural conditions, on tilled soils and sometimes with fertilizer and weed control (Hails and Morley, 2005). This may be useful for determining changes in weed infestations in crops, but not for assessing impacts on semi-natural habitats. In this paper we report experiments from both habitat types. Some previous studies have reported lower *F*_1_ hybrid fitness in semi-natural habitats (Allainguillaume *et al.*, 2006; Jørgensen *et al*., 2009; Haider *et al*., 2009), while other systems have reported *F*_1_ hybrid vigour (e.g. Hooftman *et al*., 2007) in which crop genes provide an adaptive advantage to hybrids under agricultural or similar nutrient-rich conditions (Hartman *et al*., 2013). We investigated whether the latter is also the case in *Brassica*.

In agricultural systems, a third consideration is that the persistence of hybrids needs to be assessed in the context of farming practices. OSR – *Brassica napus* – is grown in rotations with other crops across the world (Sweet *et al*., 2004; Bishnoi *et al*., 2007; Beckie *et al*., 2011; Seymour *et al*., 2012). For the UK, OSR may be used as a break crop one year in three or four (Sweet *et al*., 2004). In the intervening years *Brassica* volunteers or hybrids are generally controlled by specific herbicides and cultivation practices. However, persistence over the intervening period in the seed bank will determine hybrid and volunteer emergence in the following OSR crop.

Previous studies of crop–wild relative hybrids have addressed some of these three considerations: for example, inclusion of most life history stages in an estimate of fitness (Hooftman *et al*., 2005, 2007); and consideration of natural habitats in which a wild relative is established (Allainguillaume *et al*., 2006, 2009). To our knowledge no study has considered all three considerations simultaneously and none has considered persistence in the context of agricultural rotations. Despite several studies performed on weed seed bank dynamics and management (e.g. Gruber *et al*., 2005; Pekrun *et al*., 2006; Bohan *et al*., 2011), the relative contribution of seed bank dynamics compared with adult life stage dynamics on hybrid population persistence in agricultural systems is not well described. Furthermore, no study has analysed the influence of parental species dynamics on hybrid demography.

In this paper we address these three considerations in a study of *F*_1_ hybrids between OSR, *B. napus*, and its wild relative *B. rapa*. *B. rapa* is highly compatible with *B. napus* with a high potential of crop gene introgression (Devos *et al*., 2009; Liu *et al*., 2013) and co-occurs with it either as a weed or in road- and river-side populations in close proximity to crops (Wilkinson *et al*., 2003; Luijten *et al*., 2014). Using a combination of demographic studies and population modelling, we address the following four hypotheses. (1) Differences in vital rates between hybrids and parental species lead to changes in population fitness. (2) Inherited crop plant traits in hybrids cause low fitness compared with the wild relative in semi-natural habitats, but higher performance compared with wild relatives in agricultural conditions. (3) Crop rotations can modify the persistence time of hybrid populations in agricultural systems. (4) Interactions with the parental populations contribute strongly to the demographic dynamics of hybrid populations.

## MATERIALS AND METHODS

### Plant material, hybridization and hybrid identification

*Brassica rapa* seed was collected from semi-natural populations at three typical riparian sites in central England in July 2002, namely Bath, Radley and Wytham (Supplementary Data Fig. S1). The vegetation at each site comprised unmanaged tall herb and grass communities, with locally abundant *B. rapa.*

*F*_1_ hybrid seed was produced by growing 30 plants originating from the Radley population in the glasshouse. Following emasculation, *B. rapa* flowers were hand-pollinated by *Brassica napus* ‘Apex’ a conventional winter OSR cultivar, and then isolated against secondary pollination using sealed bags. No GM material was used in this study. After harvest, the seeds from the maternal plants were pooled. Hybridization using self-compatible species generally produces a mixture of hybrid and selfed seed, so the proportion of hybrid seed was determined using flow cytometry on 55 germinants (Supplementary Data Methods S1). The proportion of true hybrids among the plants surviving to pod production in the hybrid-sown plots (see below) was also determined using flow cytometry on leaf tissue collected *in situ*.

### Experimental design

At the three semi-natural sites in which we collected the seeds, five blocks were laid out, each containing four plots. The design enabled comparison of the performance of the three types (*B. rapa*, *B. napus* and hybrids) under undisturbed and disturbed conditions (see below). A plot comprised two parallel lines 2·5 m in length and 0·5 m apart. One line was disturbed by removing vegetation and turf 0·2 m in width and 0·05 m in depth. We sowed 50 seeds individually along the row at 0·05-m intervals. The other line was left undisturbed and 100 seeds were sown individually at 0·025-m intervals. One plot per block was sown with *B. rapa*, one with *B. napus* (OSR) ‘Apex’, one with hybrid seeds and the fourth was left unsown to estimate germination from any existing *B. rapa* seed bank. Both the disturbance treatments within plots and the sowing treatments among plots were randomly assigned. In total, 6750 seeds were sown at each site. We did not identify statistical differences between the disturbance treatments for any demographic measure (Supplementary Data Methods S2), so in the remainder of the study these data are pooled for each plant type. To assess seed survival in the seed bank, ten nylon mesh bags per species per site (i.e. 3 × 30 in total) containing 50 seeds each were buried at a depth of 10 cm.

A similar randomized block experiment was set up in an agricultural environment at the National Institute of Agricultural Botany in Cambridge (NIAB; Supplementary Data Fig. S1). Three blocks of 10 × 4 m were laid out in an arable field, which was cultivated and sown in September 2002. Each block comprised five 2·5-m lines sown with 50 seeds at 0·05-m intervals. Five types of seed were randomly assigned to the five lines: *B. napus* (OSR ‘Apex’), hybrids and *B. rapa* from each of the three field sites from which seeds were collected. A sixth line was left unsown to monitor background germination. A fourth block, being part of an accompanying experiment (unpublished), contained three repeats of the six sowing lines. Data from these three latter repeats were pooled for each plant type and comprised a fourth block in the data analysis. The field was caged against birds and irrigated, fertilized and treated with pesticides as would be an OSR crop.

### Plant vital rates

All germinants in all plots were monitored 1 week after sowing and subsequently every 3 weeks until death or seed production. In addition to (1) germination and (2) survival rates among life stages, we measured (3) fecundity. Fecundity was estimated by counting pods on all plants in the semi-natural habitats and of five random plants per line in the arable field, and by counting seeds per pod for five random pods per plant. Furthermore we measured: (4) seed viability, estimated by germination of seed samples at 20 °C on moist cotton wool following 48 h of chilling at −20 °C, with ongoing dormancy and so remaining viability of non-germinants determined by tetrazolium staining; and (5) seed bank survival, estimated using half of the buried seed bags in the following spring (overwinter survival) and the remainder in the following autumn (annual survival). The number of intact seeds was counted and viability was tested using tetrazolium staining. Staining was taken as a more reliable method than *in vitro* germination tests in which seeds may remain dormant or seedlings die before the hypocotyl is visible.

The estimation of germination rates for *B. rapa*, and of all vital rates for true hybrids, is not straightforward due to a resident *B. rapa* seed bank, and ‘hybrid seed’ being a mixture of true hybrids and *B. rapa*. This was resolved by using control line estimates of the resident seed bank, estimates of the proportion of sown seed that was truly hybrid and measures of *B. rapa* vital rates from the relevant experimental lines in a series of equations to calculate true hybrid vital rates using least squares estimation (Supplementary Data Methods S2). Where there were no significant differences in vital rates among parental species and the hybrid, they were considered to be equivalent for the following model calculations.

### Population growth rates and elasticity analyses

**Fig. 1.**
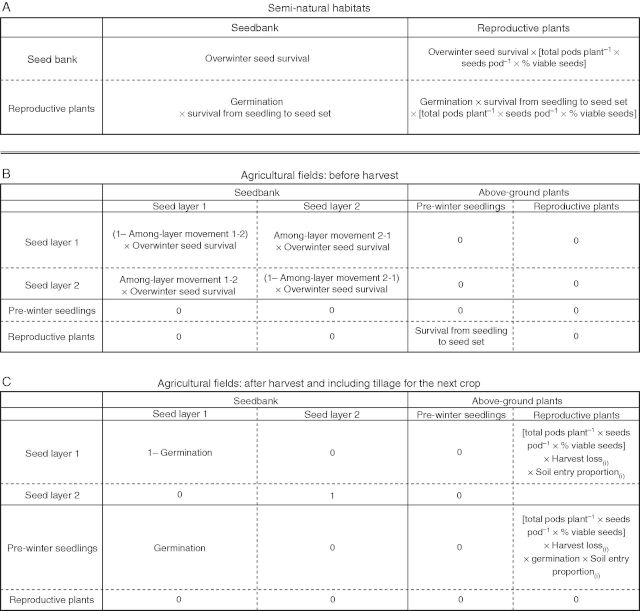
Matrices used in this study. (A) The stage-structured matrix used for semi-natural populations. (B, C) Periodic matrices for the agricultural populations for each year in the rotation. Rotation-dependent parameters (i) are the proportion of seeds entering the soil dependent on the type of tillage and harvest loss, being 5 % in years the OSR crop is planted.

We constructed density-independent, temporally explicit projection matrices of point-populations, including the transitions among life stages: seed production, adult plants and un-germinated viable seeds in the seed bank (Fig. 1). As these plants are annual, no between-year survival of adult plants was included. Transition matrices were established for each parental species and hybrids separately for each plot, combining the set of measured vital rates for that plot.

For semi-natural habitats, we used the matrix of Fig. 1A, allowing growth of hybrids and *B. rapa* in all years. Note that *B. napus* had no recorded fecundity in these semi-natural environments and therefore *λ* = 0, with only 0·3 % of seeds planted surviving until flowering and none surviving until seed-set. The predicted median for long-term population stochastic growth rate (*λ*) and the 95 % confidence intervals for *λ* were calculated from 100 000 runs, with re-sampling from plot-based sets of vital rates. For seed bank survival the average seed bank mortality among all samples per plant type was used in all calculations. Elasticity estimations were performed post hoc on the mean values among runs of the individual matrix elements (Caswell, 2000). Elasticity is a measure of the importance of each matrix element calculated as the relative change in *λ* following a change to each element.

In an agricultural habitat, *B. napus* (OSR) will be sown once every 3 or 4 years, in rotation with cereals or other broadleaved crops such as sugar beet. To simulate the effects of crop rotation, we employed periodic matrices (Caswell, 2000; Mertens *et al*., 2002). The crop rotation scenarios we simulated were those used in an earlier field trial into the implications of management of growing herbicide-tolerant OSR, and represent typical UK crop rotations (BRIGHT; Sweet *et al*., 2004). The rotations were: (1) a 3-year rotation with OSR followed by two autumn-sown cereals (e.g. wheat), i.e. the ‘BRIGHT 1’ scenario of OSR–wheat–wheat; and (2) a 4-year rotation similar to (1), but with a spring sown broadleaved crop (e.g. sugar beet) separating the two years of autumn-sown cereals, i.e. the ‘BRIGHT 3’ scenario of OSR–wheat–sugar beet–wheat. The main differences between the two scenarios are the length of the rotation and the timing of tillage. Late tillage in the spring-sown crop results in lower incorporation of *Brassica* seeds into the soil (8·5 % vs. 2 %; Gruber *et al*., 2005) and so a higher loss of seeds from the population. Rotation details are provided in Supplementary Data Methods S3. A hypothetical ‘continuous OSR rotation’ scenario was included as control, in which OSR was grown every year, allowing us to explore the effects of adding representative crop rotations on hybrid population growth (*λ*) compared with no rotations. We investigated which life-cycle elements affected *λ* the most (elasticity). Furthermore, we explored the effect of altering rotation length on *λ* and elasticities by adding hypothetical 2- and 5- to 8-year length rotations.

Separating pre- and post-harvest processes, we used a periodic matrix model with outcrossing, which is adapted from the model in Hooftman *et al*. (2007). Below we give a summary of the model, and give a detailed description of model and its parameters in Supplementary Data Methods S3. The model is written in Matlab v. 7.14.0.739 (Mathworks, Natick, MA, USA); the code can be obtained from the corresponding author. The periodic model consists of four stages all expressed in density m^−2^: seeds in two seed bank layers (shallow and deep), emerged seedlings and reproductive plants (Fig. 1). In addition to the usual demographic parameters, there are additional parameters required to account for characteristics of the agricultural habitat (Claessen *et al*., 2005): the proportion of seeds escaping harvest; the proportion of those seeds entering the soil; seed movement between the shallow and deep soil layers; and seed survival after early and spring ploughing. For parameter values see Supplementary Data Methods S3.

All three species are modelled as growing at the same time in the same field and being able to cross with each other. Following an initial pulse of *B. rapa*, representing establishment of a weed population, hybrids are formed by reciprocal outcrossing between *B. rapa* and OSR from *t* = 1 onwards. The number of hybrid seeds formed depends on the outcrossing rate, the reproductive densities of the parental species, the fecundity of the maternal species and the fecundity of hybrids itself (Hooftman *et al*., 2007). Outcrossing was set at 3 %, following Jørgensen *et al*. (2009) for a 1 : 1 mixture with 44·5 plants m^−2^. We tested for the effect of parental lines on hybrid population dynamics through new hybrid formation. The formation of hybrid seeds by the two parents was assumed to be too small to substantially influence the dynamics of the parental species

OSR is sown at the start of the rotation and is represented as emerged seedlings following immediate germination. We initiated the model with a sown OSR crop of 60 plants m^−2^, approximating a commercial density (Jørgensen *et al*., 2009). Seed banks were assumed to be empty initially. A single-pulse immigration event from *B. rapa* (e.g. as impurities in the seed) was assumed in this first year, with an initial density one order of magnitude lower (6 m^−2^). The model is run for a fixed period (*t_max*) of 100 years (BRIGHT 3 and control) and 102 years for the BRIGHT 1 scenario, allowing rotation cycles to be completed. For each run and for each species *λ* is calculated as:

(1) $$\text{λ}={\left(N_{\left(t=t\_max\right)}/N_{\left(t=\text{1}\right)}\right)}^{\text{1}/(t\_max)}$$

As for the semi-natural habitat the predicted median of the long-term stochastic population growth rate (*λ*) and the 95 % confidence intervals for *λ* are based on 100 000 runs, re-sampling from complete sets of vital rates per plot, and hence including any correlations among vital rates. The seed bank survival data were assumed to be the same as in the semi-natural habitat. The sequences of simulated matrices for the hybrid, *B. rapa* and *B. napus* were stored, allowing the same sequences to be used in the elasticity calculations below.

Elasticity (*ε*) was calculated iteratively. As hybrids and parental species are simulated to grow together, continual hybridization replenishes the seed bank of the hybrid. Therefore, changes in parental vital rates could influence the *λ* of hybrid populations. We included the indirect effect of alterations in matrix transitions of the parental populations on the *λ* of the hybrid population in our elasticity calculations. We refer to these cross plant type elasticities as ‘interactive elasticity’. To calculate such interactive elasticities we extended standard elasticity calculations (Caswell, 2000). The first component, the sensitivity (*S*, eqn 2a) we re-defined as ‘the average proportional change in *λ* of the hybrid population, caused by changing a single transition in any plant type’. To do this we elevated in turn individual matrix entries (*α_ij_*) in the (*a*) and (*b*) matrices (eqn 2a) for the hybrid, *B. rapa* and *B. napus* by 10 % following Hooftman *et al*. (2008). We calculated the proportional change in *λ* of hybrids caused by elevating each elements independently and calculated the median over 100 000 runs (sensitivity = *S*), which was subsequently normalized and *λ*-corrected to elasticity (*ε*) using standard methodology (Caswell, 2000). We averaged this elasticity over the (*a*) and (*b*) matrices (eqn 2a).

The new addition we introduce to the above standard methodology is that we now incorporate in the elasticity calculation both hybrid population dynamics itself as well as the contribution from parental dynamics to the *λ* of the hybrids via the formation of new hybrid seeds. We did this by weighting each element with the overall summed sensitivity of the full matrices (eqn 2b). Note that eqns (2a) and (2b) include normalization to ensure elasticities are comparable. For comparative analysis, we split the matrix elements into three categories adapted from Silvertown *et al*. (1996): (1) the seed bank – i.e. seed bank survival and among seed bank transitions; (2) fecundity; and (3) growth – i.e. transitions among growing plants (Supplementary Data Methods S3).

(2a) $${\text{ε}}_{ij(x)}^{h}=\frac{\left({\text{ε}}_{ij(x)}^{h(a)}+{\text{ε}}_{ij(x)}^{h(\text{b})}\right)}{\left[(\sum_{i=\text{1}}^{4}\sum_{j=\text{1}}^{4}\sum_{m=a}^{m=\text{b}}\left({\text{ε}}_{ij(x)}^{h(m)}\right)\right]}$$

with 
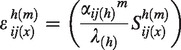
 including 
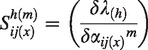

(2b) $${\hat{\varepsilon }}_{y(x)}=\theta \left[\frac{{\text{ε}}_{y(x)}^{h}}{\left({\text{ε}}_{y\text{1}(x)}^{h}+{\text{ε}}_{y\text{2}(x)}^{h}+{\text{ε}}_{y\text{3}(x)}^{h}\right)}\right]$$

with $\text{θ}=\left(S_{\left(x\right)}^{\text{tot}}/\sum^{x=h,r,n}\left(S_{\left(x\right)}^{\text{tot}}\right)\right)$ including $S_{\left(x\right)}^{\text{tot}}=\sum_{i=1}^{4}\sum_{j=1}^{4}\left(\left(S_{ij\left(x\right)}^{h\left(a\right)}+S_{ij\left(x\right)}^{h\left(b\right)}\right)/2\right)$ where $\varepsilon_{ij\left(x\right)}^{h}$^ ^= elasticity of matrix element *α_ij_* of species *x* on *λ_h_*; ${\hat{\varepsilon }}_{y\left(x\right)}$ = interactive elasticity of transition type *y* of species *x* on *λ_h_*; *m* = periodic matrix: (*a*) or (*b*); *x* = species: hybrid (*h*), *B. rapa* (*r*) and *B. napus* (*n*); $S_{ij\left(x\right)}^{h\left(m\right)}$^ ^= sensitivity of matrix element *α_ij_* in periodic matrix *m* of species *x* on *λ_h_*; *α_ij_* = stochastic matrix element with row *i* and column *j*; *λ_h_* = stochastic population growth rate of the hybrid population (eqn 1); *y* = transition type: seed bank (*y1*), growth (*y2*) and fecundity (*y3*).

## RESULTS

### Vital rates of hybrids and parental species

The hybrids went extinct in two of the three semi-natural sites, whereas *B. rapa* plants survived to seed set at all sites. Flow cytometry indicated that 65 % of the seeds resulting from the hybridization method were hybrid. The proportion of hybrids among surviving plants of the hybrid lines decreased at all semi-natural sites compared with the initial seed population (Fisher’s exact test: *P* < 0·01), indicating that hybrids had a lower survival rate than *B. rapa* (Supplementary Data Fig. S2). All following analyses that tested for differences between hybrids and *B. rapa* are for the semi-natural site in which hybrids survived (Wytham). In all semi-natural habitats, most *B. napus* (OSR) plants did not persist beyond the vegetative phase, with only seven plants flowering and none producing pods out of the 2250 *B. napus* seeds sown (Table 1); hence no full life-cycle characteristics can be given and *λ* is 0. In contrast, in the agricultural habitat the proportion of hybrids among the surviving plants in the hybrid lines did not decrease compared with that of the initial seed population, with 75 % of plants sampled being hybrid (binomial comparison; *P* = 0·63).

**Table 1.** Estimated vital rates in (a) a semi-natural (Wytham) and (b) an agricultural habitat; identical vital rates are given where differences are not significant

(a) Semi-natural habitat								
	Lambda (*λ*)	Germination (***)	Survival from seedling to adult	Survival until flowering (*)	Survival until seed set (**)	Fecundity (*P* = 0·06)	Overwinter seed survival (***)	Annual seed survival (***)
*B. rapa*	1·44	0·062	0·790	0·519	0·679	53·64	0·623	0·623
Hybrid (*B. rapa* × *B. napus*)	0·64	0·277	0·790	0·519	0·056	3·27	0·623	0·623
*B. napus*	0	0·08	0·790	0·259	0	0	0·012	0·012

(b) Agricultural habitat								
	Lambda (*λ*)^†^	Germination	Survival from seedling to adult	Survival until flowering	Survival until seed set	Pods per plant (*)	Seeds per pod (***)	Viability (***)
*B. rapa*	8·8^‡^	0·355	0·812	0·939	0·948	324	13·7	0·984
Hybrid (*B. rapa* × *B. napus*)	8·9^‡^	0·355	0·812	0·939	0·948	19·4	1·81	0·596
*B. napus*	14·9^‡^	0·355	0·812	0·939	0·948	324	23·4	0·984

† Seed survival assumed to be the same as in the semi-natural habitat.

‡ In a hypothetical 1-year rotation with continuous OSR growth; see Table 3.

*P < 0·05; **P < 0·01; ***P < 0·001.

### Semi-natural habitats

We identified significant demographic differences between the parents and hybrids at Wytham (Table 1). Hybrids had three times the germination rate (*F*_1,4_ = 45·7; *P* = 0·003) but one-twelfth the late season survival compared with their collective parent types (*F*_1,4_ = 39·5; *P* = 0·003). Fecundity was marginally significantly different between hybrids and *B. rapa*, with *B. rapa* individuals producing 15 times more seeds than hybrid individuals (*F*_1,4_ = 6·77; *P* = 0·060). No differences were found for plant survival from early to late season.

We found substantially lower seed survival in the soil for *B. napus* than for *B. rapa* and hybrids for both over-winter (*F*_2,3_ = 12·75; *P* = 0·034) and annual survival (*F*_2,5_ = 40·2; *P* = 0·001) over all sites (Table 1). *B. napus* seed survival was close to zero (1·2 %). However, *B. rapa* and hybrids did not differ, and over-winter mortality did not differ from annual mortality. This suggests that the majority of seed mortality took place over the winter.

At Wytham the mean *λ* for *B. rapa* indicated an increasing population (Table 2), although confidence intervals encompassed 1 (population stability). For the hybrid, *λ* values indicated a population decline over the full confidence range (*λ* < 1). Elasticity analysis (Table 2) showed that the overwinter seed survival rate was the most important transition for hybrid populations, whereas both fecundity and survival had high elasticities for *B. rapa*.

**Table 2.** Population growth rate (*λ*), confidence intervals and elasticities for the individual vital rates

	*B. rapa*^†^	Hybrid
Population growth rate (*λ*)	1·44	0·64
95 % Confidence interval	0·62–5·53	0·62–0·79
Elasticities		
Overwinter seed bank survival	0·40	0·99
Survival rates and fecundity^‡^	0·60	0·01

† In the habitat (Wytham) in which both hybrids and *B. rapa* survived.

‡ Survival rates and fecundity co-occur in loops only and therefore all have the same elasticity.

### Agricultural habitat

In the agricultural habitat, both parental species and hybrids survived to set seed. Hybrids had a 16 times fewer pods per plant than *B. napus* and *B. rapa* (*F*_1,7_ = 8·05; *P* = 0·025) with no difference between the two parental species (Table 1). Hybrids also had a 6·5–12 times fewer seeds per pod, with *B. napus* having the highest number (*F*_2,6_ = 84·7; *P* < 0·001). Hybrid seeds had only about half of the viability of the parental species (*F*_1,7_ = 306; *P* < 0·001), with *B. napus* and *B. rapa* seeds having equal viability (Table 1). This resulted in substantial differences in overall fecundity: 21 viable seeds per maternal plant for hybrids compared with 4368 (*B. rapa*) and 7460 (*B. napus*). We found no differences among the parental species and hybrids in germination or survival.

These vital rates resulted in a projected decline of both the *B. rapa* and hybrid population in the agricultural habitat (Table 3). However, the relatively high mean stochastic *λ* values (0·77–0·89) suggest a potential for these populations to persist for decades: a 90 % reduction in population size would take 9–20 years, depending on the rotation. The confidence intervals around these growth rates also indicate that there is some probability of population increase, albeit low.

**Table 3.** Population growth rates (*λ*), time to 90 % reduction of the population and elasticities from three rotation scenarios

	3-year rotation^†^		4-year rotation^‡^		Continuous OSR rotation^§^	
	*B. rapa*	Hybrid	*B. rapa*	Hybrid	*B. rapa*	Hybrid
Population growth rate (*λ*)	0·89	0·87	0·74	0·75	8·8	8·9
95 % Confidence interval	0·63–1·28	0·62–1·28	0·59–1·00	0·59–1·00	2·1–37·5	2·0–34·4
90 % Reduction time (years) (confidence interval)	20 (7–∞)	20 (6–∞)	9 (6–∞)	9 (6–∞)	–	–
(Interactive) elasticities						
Hybrid seed bank survival	–	0·23	–	0·17	–	0·02
Hybrid growth	–	≈ 0	–	≈ 0	–	≈ 0
Hybrid fecundity	–	≈ 0	–	≈ 0	–	0·01
Affected by *B. rapa* seed bank survival	0·43	0·71	0·61	0·80	0·05	≈ 0
Affected by *B. rapa* growth	0·03	0·03	0·03	0·02	0·04	0·14
Affected by *B. rapa* fecundity	0·54	0·02	0·37	0·01	0·91	0·83
Affected by *B. napus*	–	≈ 0	–	≈ 0	–	≈ 0

† Rotation of *B. napus* (OSR) and two autumn-sown cereals.

‡ Rotation of *B. napus* (OSR), two autumn-sown cereals with a spring-sown broadleaved crop in between.

§ Hypothetical continuous OSR cultivation without other crops.

**Fig. 2.**
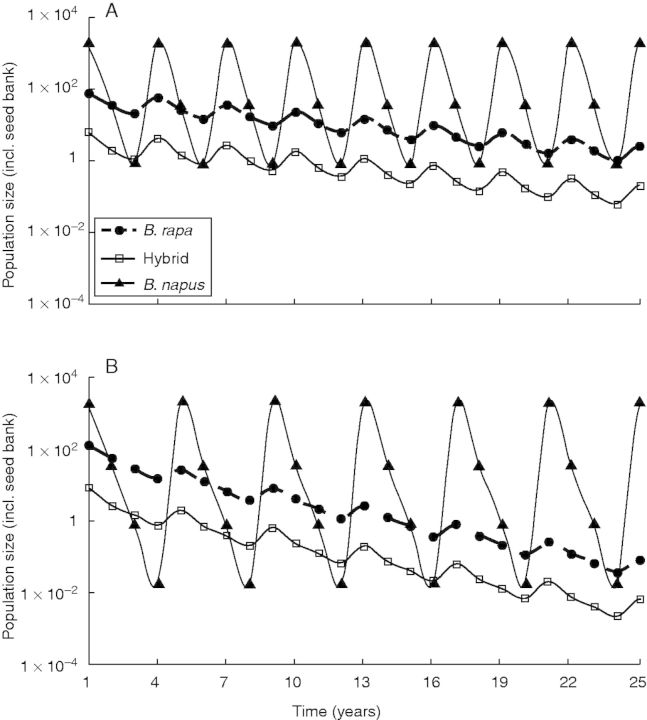
Population sizes in crop rotation scenarios for *B. rapa*, *B. napus* and the hybrid, with hybrids formed by outcrossing between the parents from *t* = 1 onwards. (A) A 3-year rotation of oilseed rape (OSR) and two autumn sown cereals (BRIGHT 1). (B) A 4-year rotation of OSR, an autumn sown cereal, a broadleaved crop and an autumn sown cereal (BRIGHT 3). The difference between both scenarios is the length of the period between planting an OSR crop. Populations start from emerged seedlings only, mimicking an immigration event and first time OSR cultivation.

The parental species and hybrid all showed density cycles caused by reproduction – or sowing for OSR – occurring only in years in which OSR was the crop (‘OSR years’; Fig. 2). In intervening years, populations of *B. rapa* and hybrids were reduced through seed mortality in the soil and death of plants following germination from the upper soil layer. As a consequence, *λ* values were lower under the 4-year rotation scenario because of the longer interval between years in which seed set was possible (see extended analyses below). Note that *B. napus* is sown as a crop in these simulations, and hence has a *λ* of 1.

Hybrid growth rates followed the growth rates of *B. rapa* closely, with similar confidence intervals (Table 3). The mechanism here is that the low fecundity of hybrids is partially compensated for by new *F*_1_ hybrid seeds in OSR years. This is confirmed by the interactive elasticities (Table 3); the hybrid population growth rate was mostly driven by the dynamics of the wild parent. The foremost ‘interactive elasticity’ of the hybrid *λ* was in the seed bank transitions of *B. rapa*. This suggests that the hybrid population is largely dependent on the ability of the wild parent to survive in the seed bank, complete its life cycle in OSR years and cross with OSR to form new *F*_1_ hybrid offspring. This contrasts with the dynamics of *B. rapa* itself for which elasticity is balanced among seed bank and fecundity vital rates.

By contrast, in the hypothetical control scenario of continuous OSR production the *λ* values of both *B. rapa* and hybrids were mostly sensitive to above-ground dynamics, especially fecundity of *B. rapa*. This result indicates that the importance of the seed bank – outlined above – is caused by agricultural rotations in which germinants are controlled in non-OSR years.

**Fig. 3.**
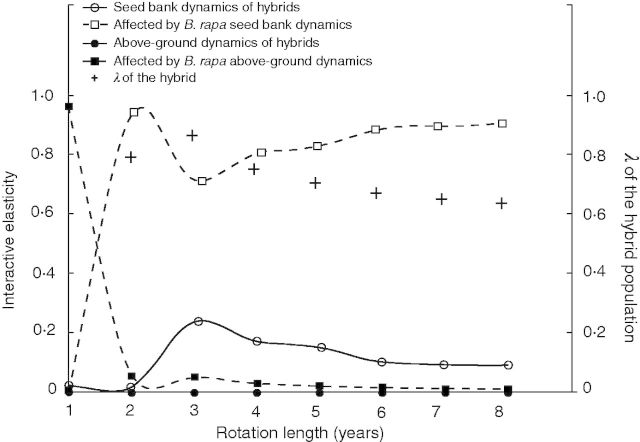
Simulated changes in ‘interactive’ elasticity of hybrid and *B. rapa* above- or below-ground vital rates with hypothetical shortened or lengthened rotations. The proportional importance in influencing the growth rate of the hybrid population is shown for the matrix elements that denote either above- or below-ground dynamics of the hybrid population itself or of its wild parent through replenishment. The influence of *B. napus* on the hybrid population growth rate is not shown as its elasticity ≈ 0 for all elements.

We examined the effect of rotation length further by removing or adding non-OSR crops into the rotations (Fig. 3). As the length of the rotation increases high elasticity of above-ground stages in the continuous OSR scenario is replaced by high elasticity of *B. rapa* below-ground seed bank transitions (≈0·9), with a retained lower elasticity of the seed bank transitions of the hybrids itself (≈0·1). The results are identical for the two BRIGHT scenarios. This is because no sequential combinations of the spring-sown broadleaved crop and OSR are feasible, which would have differentiated between both scenarios based on the differing proportion of seeds incorporated in the soil between autumn- and spring-sown crops (Supplementary Data Methods S3).

## DISCUSSION

We found substantial fitness differences between the *F*_1_ hybrid of *Brassica rapa × Brassica napus* (OSR) and both parental species. Furthermore, the relative contributions of different life stages to fitness differences depend upon habitat type, i.e. semi-natural or agricultural, and type of rotation. Combining manipulative experiments and modelling, as we have done here, strongly facilitates understanding of the population dynamics of these systems. Better understanding is an important step in managing hybrid populations and designing mitigation strategies against environmental damage from the presence of hybrids. Such strategy considerations could also include testing for the stability of introgression through recombination and for the maintenance of introgressed genes in the recipient populations (Devos *et al*., 2009; Ellstrand *et al*., 2013; Garnier *et al*., 2014).

### Hypotheses

Three of our four hypotheses were substantiated. Our first hypothesis was that we would detect differences in vital rates, and ultimately in the fitness – i.e. the population growth rate – of hybrids compared with the parental species. This was indeed the case, with hybrids having lower fitness or being intermediate in fitness between the two parental species in the semi-natural habitat in which hybrids survived, as is generally expected for *F*_1_ hybrids (Burke and Arnold, 2001). The hybrid growth rate indicated population decrease (*λ* < 1) compared with *B. rapa* which had a positive population growth rate, and OSR which had a growth rate of zero. In the agricultural habitat both hybrids and *B. rapa* had only potential population growth rates of *λ* > 1 when rotations were not considered. When including rotations mean *λ* dropped below 1. These findings of a low *F*_1_ hybrid fitness are in line with other results for *Brassica* hybrids, e.g. as reported by Allainguillaume *et al.* (2006), Jørgensen *et al.* (2009) and Haider *et al.* (2009). We acknowledge that there could be substantial vital rate differences among OSR cultivars (Lutman *et al*., 2003, 2005); we consider ‘Apex’ a representative example open-pollinated cultivar as it has been cultivated for many years in the UK and used in many studies such as Sweet *et al.* (2004) and Lutman *et al.* (2005).

Our second hypothesis was not substantiated. We suggested that inherited crop plant traits would lead to a growth rate for hybrids below that of *B. rapa* in semi-natural habitats, but higher than *B. rapa* in agricultural conditions because of pre-adaptation of crop genes to the crop environment. However, hybrids performed worse than *B. rapa* because of low fecundity in both semi-natural and agricultural conditions. Furthermore, hybrid fitness differed substantially across sites, being zero at two of the three semi-natural sites. Our elasticity analysis showed that the demographic dynamics of hybrids were very different from those of *B. rapa*: hybrid dynamics were largely dependent on the seed bank in both semi-natural and agricultural habitats.

Our third hypothesis was substantiated. We suggested that rotations could modify the projected persistence of hybrids in agricultural systems as has been found for weed species (Bohan *et al*., 2011; Gulden *et al*., 2011). In rotations including non-OSR years, i.e. years without the possibility for *Brassica* seed set, growth rates indicated population decreases (*λ* < 1) and the dominant matrix elasticities switched from above-ground dynamics to seed bank survival. However, hybrid populations could still persist for >20 years under the assumptions of our model, parameterized using our field data. Comparing the 3- and 4-year rotations, the declines of *B. rapa* and hybrids were more precipitous in the latter. Further examination showed that because the seed bank of both the wild *Brassica* and hybrids is only replenished when OSR is grown, under a longer rotation more seeds have died by the time a new OSR crop is sown. The rate of hybrid decline was little affected by its fecundity, but was determined by hybrid seed bank survival and, most importantly, by the seed bank dynamics of *B. rapa*. This last point confirms our last hypothesis. Interactions with the parental populations, in terms of continual hybridization events, strongly affect the population dynamics and growth rate of hybrid populations.

### The role of the seed bank

Overall, in agricultural habitats the dynamics of the seed bank proved to be the dominant driver of the hybrid life cycle. This importantly includes the seed bank dynamics of the wild parents in a situation in which *F*_1_ hybrids can be formed continually and hybrid fecundity is low. OSR is one of many crops grown in rotation. The resulting periodicity of rotations means the seed bank and its accompanying management is of prime importance for many weeds (Bohan *et al*., 2011; Gulden *et al*., 2011), including weedy hybrids as shown here. Our elasticity analysis revealed that in *Brassica* the seed-bank-related transitions are the most influential and contribute most to the differences in population persistence, a conclusion which we share with Claessen *et al.* (2005). In both habitat types the hybrid population was estimated to be in decline but hybrids could persist for decades in agricultural habitats through survival in the seed bank combined with periodic continual hybridization events.

In considering fitness of hybrids in the Brassicaceae and other families, fecundity is the most frequently studied trait using either GM or conventional plants as model systems (e.g. Snow *et al*., 1999; Allainguillaume *et al*., 2006; Jørgensen *et al*., 2009), with other traits much more rarely studied. However, it seems it is those poorly studied traits, such as seed bank survival, that are the most important to populations in both semi-natural and agricultural settings. This result strengthens the call for better insights into interactions between the seed bank, the environment and management (Gruber *et al*., 2005; Bohan *et al*., 2011; Gulden *et al*., 2011).

The explicit novel finding from this study is that, particularly in an agricultural setting, the seed bank survival of the wild relative (*B. rapa*) is the most influential for the hybrid growth rate. The factors influencing these demographic transitions (number of seeds escaping harvest, movement between layers, etc.) are influenced by management and would benefit from future experimental study. However, we expect that in cases where *Brassica* hybrid fecundity is restored in backcross generations (see, for example, Jørgensen *et al*., 2009; Snow *et al*., 2010) the dependence on the parental species would decrease, whereas higher outcrossing rates would further increase dependence on the parental seed bank dynamics.

### Introgression beyond *F*_1_ hybrids

We made the simplifying assumption that the fitness of hybrids is well described by the low fitness of the *F*_1_ generation, but recognize that our conclusions are dependent upon this assumption. The subsequent process of backcrossing was not studied here but has been studied experimentally for *B. rapa* × *B. napus* hybrids by Jorgensen and co-workers (summarized by Jørgensen *et al*., 2009) and recently by Liu *et al.* (2013). Those studies show that *Brassica* hybrids have highly variable fitness (Devos *et al*., 2009; Jørgensen *et al*., 2009). Furthermore, several studies suggest that *F*_1_ hybrids might have reduced fitness compared with later backcrosses (Burke and Arnold, 2001; Snow *et al*., 2010; Yang *et al*., 2011) and fitness could bounce back in later generations towards that of the wild parent (Liu *et al*., 2013; Cao *et al*., 2014). Therefore, our fitness estimates of *Brassica* hybrids are likely at the lower end of what might happen in reality. Despite these issues, low fecundity of hybrids would result in very few such backcrosses and the hybrid population being dominated by newly created *F*_1_ hybrids during OSR crop years. Moreover, other factors that we did not study can arrest development of further generations; for example, male fitness in later generations could be impaired, flower morphologies become incompatible or flowering seasons become asynchronous (Norris *et al*., 2004; Warwick *et al*., 2004; Jørgensen *et al*., 2009). Furthermore, chromosomal selection might be biased (de Jong and Hesse, 2012), resulting in unequal segregation of traits that affect fitness, selecting against later generation hybrids.

As long as an additional transgene in hybrids is not subject to its intended stress benefit outside of the crop, such as diseases or insects, its introgression rate could be based on neutral segregation ratios across generations. However, in those cases where these stresses remain, such as herbicide spraying, tolerant hybrids could have an additional benefit and so increase their presence in the population (Devos *et al*., 2012; Gressel, 2014). Transgene presence may also interact with the hybridization process; a transgene will be subject to positive or negative genetic hitchhiking based on its position on the chromosome as well as affecting the likelihood of selection of that chromosomal segment (Hartman *et al*., 2013). Furthermore, the transgene could incur fitness costs or benefits to plant growth (Snow *et al*., 1999; Hails and Morley, 2005; de Jong and Rong, 2013). Therefore, for an environmental risk assessment of GM crops, the potential for survival of hybrids based on population dynamics is a first step, which has to be complemented by the further impact of transgenic traits on hybrid survival. Garnier *et al*. (2014) provide a modelling framework that could be used.

## CONCLUSIONS

In this study we assessed which aspects of the life cycle of hybrids and their parents were of principal importance for the persistence of hybrids. We found that changes in seed bank survival rate may have the greatest impact on long-term persistence of hybrid populations. We suggest that more research should focus on traits that alter seed persistence dynamics or seed dormancy. Similarly, changes in management can interact with changes in fitness: the timing and depth of tillage are known to influence the level of dormancy and survival of seeds and thus the longevity of the wild relative or hybrid seed bank (Gruber *et al*., 2005; Pekrun *et al*., 2005, 2006). Therefore, there may be the potential for changes in the timing and depth of tillage operations to control hybrid populations. These suggestions are based on the assumption that hybrid populations are undesirable in and among crop fields and their presence needs to be managed (Snow *et al*., 2005; Chandler and Dunwell, 2008; Schierenbeck and Ellstrand, 2009).

For hybrids with low fecundity, as long as a new inherited (GM) trait does not increase fecundity, local risk mitigation could additionally focus on the wild relative. To manage hybrid presence, selective herbicide treatments could be effective even in those cases in which herbicide-tolerant *F*_1_ hybrids occur, as herbicides would still kill the wild relative. Such management would reduce hybrid populations indirectly by blocking seed bank replenishment. We stress that the role crop rotations play in hybrid population persistence deserves greater attention as they strongly influence the dynamics of both hybrids and wild relatives.

*B. napus*, both as a crop (OSR) and as a feral plant, is a particularly pertinent case study, due to the regular presence of wild relatives (*B. rapa*) in both semi-natural habitats and as crop weeds (Wilkinson *et al*., 2003; Luijten *et al*., 2014). We emphasize, however, that the principles and approach presented here have a broader applicability elsewhere, and the techniques applied to study dynamics in semi-natural habitats can reveal new insights when applied to crop systems.

## SUPPLEMENTARY DATA

Supplementary data are available online at www.aob.oxfordjournals.org and consist of the following. Fig. S1: Map of the experimental locations and seed collections of *B. rapa*. Fig. S2: Proportion of hybrids confirmed by flow cytometry from plants grown from the original seed sample under controlled conditions compared with plants sampled at seed set in the experimental sites. Methods S1: Hybrid identification. Methods S2: Vital rates and full among-line statistics Methods S3: Periodic matrix model.
